# Cohort profile: cholangiocarcinoma screening and care program (CASCAP)

**DOI:** 10.1186/s12885-015-1475-7

**Published:** 2015-06-09

**Authors:** Narong Khuntikeo, Nittaya Chamadol, Puangrat Yongvanit, Watcharin Loilome, Nisana Namwat, Paiboon Sithithaworn, Ross H. Andrews, Trevor N. Petney, Supannee Promthet, Kavin Thinkhamrop, Chaiwat Tawarungruang, Bandit Thinkhamrop, on behalf of the CASCAP investigators

**Affiliations:** 1 Cholangiocarcinoma Screening and Care Program (CASCAP), Khon Kaen University, Khon Kaen, 40002 Thailand; 2 Department of Surgery, Faculty of Medicine, Khon Kaen University, Khon Kaen, Thailand; 3 Department of Radiology, Faculty of Medicine, Khon Kaen University, Khon Kaen, Thailand; 4 Department of Biochemistry, Faculty of Medicine, Khon Kaen University, Khon Kaen, Thailand; 5 Department of Parasitology, Faculty of Medicine, Khon Kaen University, Khon Kaen, Thailand; 6 Faculty of Medicine, St Mary’s Campus, Imperial College, London, United Kingdom; 7 Department of Ecology and Parasitology, Karlsruhe Institute of Technology, 76131 Karlsruhe, Germany; 8 Department of Epidemiology, Faculty of Public Health, Khon Kaen University, Khon Kaen, Thailand; 9 Data Management and Statistical Analysis Center (DAMASAC), Faculty of Public Health, Khon Kaen University, Khon Kaen, Thailand

**Keywords:** Bile duct cancer, Cholangiocarcinoma, Cancer screening, Cancer registry, Cohort study

## Abstract

**Background:**

Cholangiocarcinoma (CCA) is an extremely aggressive cancer that is usually fatal. Although globally morbidity and mortality are increasing, knowledge of the disease remains limited. The Mekong region of Southeast Asia, and particularly the northeast of Thailand, has by far the highest incidence of CCA worldwide with 135.4 per 100,000 among males and 43.0 per 100,000 among females being reported in Khon Kaen Province. Most patients are first seen during late stage disease with 5-year survival being less than 10 %. Starting in 1984, control and prevention strategies have been focused on health education. Although early detection can substantially increase 5-year survival, there are currently no strategies to increase early diagnosis.

**Methods/design:**

The Cholangiocarcinoma Screening and Care Program (CASCAP) is a prospective cohort study comprising two cohorts- the screening and the patient cohorts. For the screening cohort, ultrasound examination will be carried out regularly at least annually to determine whether there is current bile duct and/or liver pathology so that the optimal screening program for early diagnosis can be established. This cohort is expected to include at least 150,000 individuals coming from high-risk areas for CCA. For the patient cohort, it is estimated that about 25,000 CCA patients will be included during the 5-year recruitment period. All CCA patients will be treated according to routine clinical care and followed so that effective surgical treatment can be formulated. This cohort is indeed a conventional cancer registry. Thus, CASCAP is an ongoing project in which the number of participants changes dynamically.

**Discussions:**

This is the first project on CCA that involves screening the at risk population at the community level. At the time of preparing this report, a total of 85,927 individuals have been enrolled in the screening cohort, 55.0 % of whom have already undergone ultrasound screening, and 2661 CCA cases have been enrolled in the patient cohort. Among the participants of the screening, whose mean age was 53.8 ± 9.8 years, 55.6 % were female, 77.5 % attained primary school as the highest level of education, 79.9 % were farmers, 29.9 %, reported having relatives with CCA, 89.1 % had eaten uncooked fish, and 42.2 % of those who had been tested for liver fluke were found to be infected.

## Background

Cholangiocarcinoma (CCA) is a biliary duct cancer that commonly invades surrounding liver tissue [1]. It represents the second most common primary hepatic malignancy, comprising 30 % of primary hepatic tumors worldwide, and has very high mortality rates, primarily due to late detection [2, 3]. Both intra- and extrahepatic forms occur [1, 4]. Incidence appears to be increasing worldwide including in Europe and the USA [4–6], although this is dependent of geographical locality. In the USA, the annual incidence was 2000–3000 cases [7]. In Europe, the rates of primary liver cancer have declined, while those for intrahepatic CCA have increased throughout the European Union by 9 % from1996 to 2008. Recent developments in CCA staging have been based mainly on hepatocellular carcinoma and have failed to stratify CCA patients adequately and appropriately, particularly with respect to tumor size [8].

The area with by far the highest incidence of CCA worldwide is along lower Mekong area of Southeast Asia. This is the distributional area of the liver fluke *Opisthorchis viverrini*, which is classified as a class one carcinogen and is a major risk factor for developing CCA in this area [9]. Infection occurs via the consumption of raw or undercooked freshwater fish belonging to the carp family [10]. The countries bordering the Mekong have an estimated 90 million people at risk of infection with an estimated 10 million being infected, although reliable data are currently only available from Thailand and, in part, Lao PDR [11, 12], while there are currently no reliable data for Cambodia and southern Vietnam.

Treatment options include liver resection or transplantation and radiation therapy [13–16]. Although, the general prognosis is poor, with low 5-year survival rates of usually less than 20 % [17–21], a 65 % cure rate may be achieved in some cases with perihilar cholangiocarcinoma that were treated with neo-adjuvant therapy followed up by liver surgery [22]. There is increasing evidence that early diagnosis leading to liver resection can substantially improved the patient’s chances of survival and quality of life [17, 23, 24]. However, strategies for determining the main population at risk and thus for developing an effective screening program remain to be developed.

Thailand has an estimated eight million infected individuals [25], with an incidence reaching up to 87.7 per 100,000 in males and 36.3 per 100,000 in females [26], in the northeast of the country which has the highest incidence of CCA worldwide [27]. Although there are various estimates of the total number of cases occurring annually, a conservative estimate suggests that 20,000 or more deaths/year occur due to intra- and extra-hepatic CCA in the northeast of Thailand alone [28]. This mortality is directly related to difficulties in diagnosing the disease at an early stage when surgical cure is possible. Given the poor prognosis due to late-stage discovery of the disease, and the fact that CCA commonly manifests at or after the age of 40, with males being more commonly infected than females, not only is the patient directly affected, but the family for which he or she is responsible is also liable to socio-economic hardship [29].

The Cholangiocarcinoma Screening and Care Program (CASCAP) was developed at the Medical Faculty of Khon Kaen University in cooperation with the Cholangiocarcinoma Foundation, Thailand. It represents the most detailed and comprehensive study of the application and optimization of screening methods for the early diagnosis of CCA combined with treatment and follow-up worldwide. It includes a sophisticated data collection and analysis system that can be used to determine government policy for the treatment and control of CCA not only within Thailand but also in other Mekong countries.

CASCAP has multiple aims in addition to developing a strategy for diagnosing early-stage CCA. It will increase awareness of CCA in the at risk population and by doing so reduce the costs of screening. It will reduce the incidence of CCA by determining which individuals are at high risk based on precancerous pathology and following them up. As CASCAP data are collected both longitudinally as well as a cross-sectionally, progressive changes in the bile duct and liver of individuals can be monitored. Once diagnosed, patients will receive the best available treatment. They will then be followed-up and provided with the best supportive care and clinical assessment until the end of life.

Although the CASCAP program is health care oriented, the data collected represent a massive research database for scientific monitoring and evaluation, and in particular the assessment of long-term changes in the liver and the bile duct, the rate of early detection, and long-term clinical outcome of the patients in response to various medical interventions.

The procedures defined by CASCAP are ultimately aimed at being adopted as part of routine health-care practice. All core data items are part of the routine data collection at all cancer hospitals in the northeastern region of Thailand and later throughout the Mekong region. CASCAP serves as an innovative and comprehensive approach to combat CCA in the region.

## Methods/design

### Design overview

**Fig. 1:**
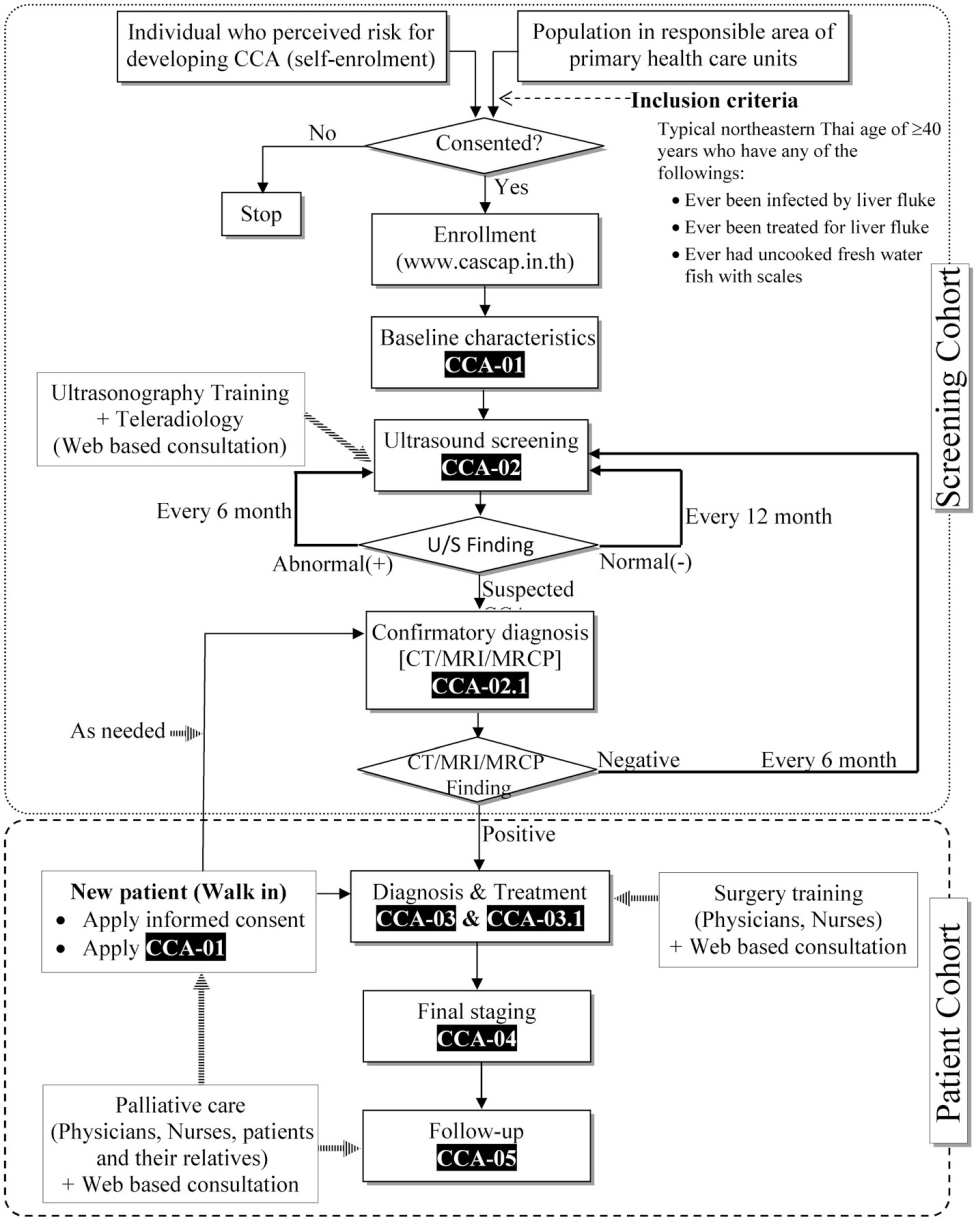
Workflow of the Cholangiocarcinoma Screening and Car Program (CASCAP). CCA = cholangiocarcinoma, U/S = ultrasonography, CT = computed tomography, MRI = magnetic resonance imaging, MRCP = magnetic resonance cholangiopancreatography

CASCAP is a prospective cohort study. Two cohorts have been defined, one for determining who should be screened and the other for patients diagnosed as having CCA. The screening cohort, characterized in the upper zone of Fig. 1, is expected to include at least 150,000 individuals coming from high-risk areas for CCA, including the self-enrolment of persons who feel that they are in danger of developing CCA. The inclusion criteria include all endemic northeastern Thais of 40 years or over with any of the following: ever been infected by or treated for liver flukes or known to have eaten uncooked freshwater fish with scales. Once consent has been obtained, the participants will be enrolled in the program and their baseline information collected. If CCA has been diagnosed, the individual will automatically be moved to the second, patient cohort, characterized in the lower zone of Fig. 1.

After the baseline characteristics have been recorded, an ultrasound examination will be carried out (the upper zone of Fig. 1) to determine whether there is current bile duct and/or liver pathology. Patients with liver mass or bile duct dilatation will be directed to confirmatory diagnostic tests and, if CCA is confirmed, they will be transferred to the patient cohort (the lower zone of Fig. 1).

The patient cohort will include all CCA patients diagnosed in the region over a 5-year duration, with an estimated number of 5000 histologically proven new cases per year or 25,000 in the five-year recruitment period. It will include individuals diagnosed from the screening cohort, as well as new patients diagnosed at the nine tertiary-care hospitals participating in the CASCAP study. After diagnosis, the treatment regime will be determined and recorded. These patients will be treated according to the routine care procedures of the hospitals. Follow-up treatment will be recorded. Patients will be followed-up until the end of life.

### Follow-up schedule

For the screening cohort, participants will undergo ultrasonography every 12 months if the findings are negative, and every 6 months if the diagnosis is positive for either periductal fibrosis of the bile duct, or fatty liver, or cirrhosis. They will be immediately referred to CT or MRI if they have liver mass or bile duct dilatation.

For the patient cohort, treatment follow-up will be based on local routine care every 3 month for the first year and every 6 month thereafter. CASCAP will follow up all patients every year to update information on their health.

### Data collection

There are six data collection forms indicated as CCA-01 to CCA-05 according to each phase as shown in Fig. 1, with details of the parameters being collected shown in Table 1. These include the Demographic Information and Enrollment Form (CCA-01), Ultrasound Form (CCA-02), Confirmatory Diagnosis Form (CCA-02.1), Diagnosis and Treatment at the 1st visit (CCA-03), Follow-up Treatment Form (CCA-03.1), Final Staging Diagnosis (CCA-04), and Post Operation Follow-up Form (CCA-05).

**Table 1:** Data being collected

**CCA** **-01:** **Demographic information form:** **Enrollment**
1. Place of resident
2. Date of birth
3. Gender
4. Education
5. Occupation
6. Number of stool examination for liver fluke infection in the past
7. Being ever had found to be infected by liver fluke
8. Number of occasions being treated for liver fluke infection
9. Relatives diagnosed with cholangiocarcinoma
10. Cigarette smoking
11. Alcohol drinking
12. Being ever had chronic alcoholic toxicity
13. Being ever had eaten uncooked or fermented fish (specifically, fresh water with scales)
14. Underlying diseases
**CCA-02: Ultrasound form**
15. Liver
15.1) Parenchymal ECHO (Normal, Abnormal)
15.2) Fatty liver (Mild, Moderate, Severe)
15.3) Periductal fibrosis (PDF1, PDF2, PDF3)
15.4) Cirrhosis
15.5) Liver Mass (No, Single Mass, Multiple Masses)
15.6) Liver mass characteristics (High echo, Low echo, Mixed echo, Liver cyst)
15.7) Liver mass size
15.8) Liver mass side (left, right)
15.9) Dilated Bile Duct (No dilated duct, Right lobe, Left lobe, Common bile duct)
16. Gallbladder
16.1) Gallbladder findings (Normal, Abnormal)
16.2) Gallbladder wall thickening (Focal, Diffuse) and size
16.3) Gallbladder polyp (Single, Multiple) and size
16.4) Gallbladder mass (Single, Multiple) and size
16.5) Gallstone (None, Single, Multiple)
16.6) Being post cholecystectomy
17. Kidney
17.1) Kidney (Normal, Abnormal)
17.2) Renal cyst (None, Right, Left)
17.3) Parenchymal change (None, Right, Left)
17.4) Renal stone (None, without hydronephrosis, with hydronephrosis)
17.5) Renal stone (None, Right, Left)
17.6) Being post nephrectomy
18. Other Finding (Ascites, Splenomegaly, Others)
**CCA-02.1: Confirmatory diagnosis form**
19. Mode of confirmatory Diagnosis (CT, MRI, Others)
20. Finding and location of tumor in the bile duct (Normal, Intrahepatic, Perihilar, Distal, Other diseases)
21. Side of intrahepatic CCA, if any (Right lobe, Left lobe)
22. Type of perihilar CCA (BC 1, BC 2, BC 3a, BC 3b, BC 4)
23. Tumor morphology
23.1) Mass forming (nodular) and size
23.2) Periductal infiltrating type
23.3) Intraductal type and size
23.4) Mixed type
24. Hepatic artery (Normal, Encasement)
25. Hepatic vein (Normal, Encasement)
26. Portal vein (Normal, Encasement)
27. Lymph node (Normal, Positive node along hepatoduodenal ligament, Positive at others nodes)
28. Adjacent organ involvement / Distant metastases (No, Yes)
29. Type of organ involvement, if any (Lymph node, Lungs and pleura, Bone, Brain, Peritoneum, Others)
**CCA-03: Diagnosis and treatment at the 1st visit**
30. Surgical Treatment (Done, Not done)
31. Tumor site (Intrahepatic CCA, Perihilar CCA, Distal CCA, Other diseases)
32. Clinical Staging (TNM)
33. Treatment protocol being implemented
33.1) Surgery (Liver resection, Hilar resection, Bypass, Exploratory laparotomy +/− biopsy, Whipple’s operation)
33.1) Chemotherapy (Adjuvant, Palliative)
33.1) PTBD (Pre-op therapy, Palliative)
33.1) Endoscopic Stent (Pre-op therapy, Palliative)
33.1) Medication Treatment (IV, Antibiotics, Others)
34. Best supportive Treatment (Yes, No)
35. Results (Death, Discharged, Referred to other hospitals)
**CCA-03.1: Follow-up treatment form**
36. Treatment protocol being implemented
36.1) Surgery (Liver resection, Hilar resection, Bypass, Exploratory laparotomy +/− biopsy, Whipple’s operation)
36.1) Chemotherapy (Adjuvant, Palliative)
36.1) PTBD (Pre-op therapy, Palliative)
36.1) Endoscopic Stent (Pre-op therapy, Palliative)
36.1) Medication Treatment (IV, Antibiotics, Others)
37. Best supportive Treatment (Yes, No)
38. Results (Death, Discharged, Referred to other hospitals)
**CCA-04: Final staging diagnosis**
39. Tumor site (Intrahepatic bile duct (CCA, C221), Perihilar (CCA, C240), Distal (CCA, C241), Others, non-specified)
40. Marginal status (R0-free margin, R1-not free margin, microscopic, R2-not free margin, gross finding)
41. Lymph node status (N0-no metastasis, N1-metastasis hepatoduodenal node, node 8 or 12, N2-metastasis aortocarval, node 9, 13, 16)
42. Histology (Non papillary, Papillary non invasive, Papillary invasive, Other type)
43. CCA Staging (Stage 0, I, II, IIIA,IIIB, IVA, IVB, Unknown)
44. Metastasis (No data, None, Lymph node, Lungs and pleura, Bone, Brain, Peritoneum, Liver, Others)
**CCA-05: Post operation follow-up form**
45. Date and mode of follow-up (By hospital visit, By phone call)
46. Status of the patient (Health, Recurrent, Progress, Withdrawn consent, Loss to follow-up >3 months after the appointment, Dead and Cause of dead)
47. Being treated at other hospitals prior to this visit (Yes, No)
48. Co-morbidity (None, Diabetes, Hypertension, Heart disease, Others)
49. Complications (None, Cholangitis, Liver failure, Pancreatitis, 50. Renal failure, Pleural effusion, Intra abdominal bleeding, Wound infection, Ascites, Prolonged bile leakage, Others

### Statistical methods

With a targeted sample size of 150,000 participants to be screened for CCA, we expect to detect early stage CCA at a rate as low as one per 1000 with a precision of no more than ±0.16 per 1000. A minimum of 1500 early stage CCA patients is sufficient to demonstrate the efficacy of the various treatment protocols provided to this cohort compared to the patient group at late stage detection, as well as comparing across the treatment protocols. At the end of the recruitment period, we expect to achieve a total sample size of 25,000 patients with some stage of the disease. This sample size would allow us to calculate a hazard ratio (HR) of at least 1.5, with a power of greater than 99 % for progression-free survival comparing either across treatment protocols or disease stages. This was estimated based on an assumption of the overall anticipated event rate of 0.5 with a correlation among covariates of 0.1 and based on using cox regression as the statistical method.

For additional statistical analysis, we will estimate the rate of CCA together with its 95 % confidence interval (95%CI), particularly for early stage CCA, as well as the corresponding rate for each relevant subgroup. Survival analysis will be used to obtain survival profiles, including time to detection, time to disease recurrence or death, survival probability at 1–5 years, and hazard ratios with their 95%CIs. For these purposes, Kaplan-Meier methods and cox regression will be used. All analyses will be performed using Stata version 13 (StataCorp, College Station, TX). Significance level is set as 0.05 and all statistical tests will be two-sided.

### Ethics and good clinical practice

This study will be performed according to the principles of Good Clinical Practice [Chapter 2 of the ICH Harmonized Tripartite Guideline for Good Clinical Practice (GCP)], the declaration of Helsinki, and national laws and regulations about clinical studies. The CASCAP was approved by the Khon Kaen University Ethics Committee for Human Research on February 9, 2013. The first participant was enrolled on March 5, 2013.

## Discussions

### Cohort profiles

CASCAP is an ongoing project in which the number of participants changes dynamically. For the purpose of this preliminary report, the data at the completion of the first year of the project were analyzed and the characteristic of participants at baseline were reported.

A total of 85,927 individuals were enrolled with a mean age of 53.8 ± 9.8 years of whom 55.6 % were female (Table 2). About three quarters, 77.5 %, had attained primary school as the highest level of education, and 79.9 % were farmers. More than half, 55.9 % had previously been tested for liver fluke infection. Among the 47,258 participants who had been tested, 42.2 % were found to be infected. About a quarter, 29.9 %, reported they have relatives with CCA and 89.1 % had previously eaten uncooked or fermented fish (specifically, freshwater fish with scales).

**Table 2:** Demographic and baseline information collected on enrollment

Characteristic on enrolment	n (%)
Gender	
Male	38,066 (44.4)
Female	47,739 (55.6)
Age (years)	
40–49	33,595 (39.1)
50–59	29,292 (34.1)
60–69	16,724 (19.5)
70+	6316 (7.3)
Mean (Standard deviation)	53.82 (9.77)
Median (Minimum : Maximum)	52 (40 : 100)
Educational attainment	
No formal education	1134 (1.3)
Primary school	66,000 (77.5)
Secondary school level 1	6194 (7.3)
Secondary school level 2	6639 (7.8)
College	1127 (1.3)
Bachelors degree	3154 (3.7)
Masters degree or higher	931 (1.1)
Occupation	
Unemployed	2787 (3.3)
Farmer	68,060 (79.9)
Labor	5378 (6.3)
Self-employed	2594 (3.0)
Government/state enterprise	4198 (4.9)
Others	2189 (2.6)
Number of occasions of fecal examination for liver fluke infection	
0	31,620 (37.2)
1	33,667 (39.6)
2	9806 (11.5)
3	3033 (3.6)
More than 3	3504 (4.1)
Cannot remember	3426 (4.0)
Being found to be infected by liver fluke	
Never tested	32,121 (38.0)
Tested but negative	27,296 (32.3)
Tested and positive	19,962 (23.6)
Cannot remember	5161 (6.1)
Rate of liver fluke infection	47,258 (42.2)
Relatives diagnosed with cholangiocarcinoma	
None	59,562 (70.1)
Yes	25,445 (29.9)
Ever eaten uncooked or fermented fish (specifically, freshwater with scales)	
No	9271 (10.9)
Yes, current or previous	75,816 (89.1)

At the time of preparing this report, there were 47,285 (55.0 %) participants who had undergone ultrasonographic screening and 2661 CCA cases have been enrolled in the patient cohort (data not shown).

### Strengths and weaknesses

Strengths of the CASCAP study include the novelty and uniqueness of the project. This is the first project on CCA that involves screening the at risk population at the community level. The initial screening involved ultrasonography with the images being evaluated independently by at least two trained radiologists.

The project incorporates two cohorts which provided the largest pool of CCA risk participants and CCA patients worldwide. In addition, it allows for long term monitoring and the ability to discovering rare events, such as the development of CCA. For the screening cohort, participants were recruited at the community level by their local health service providers, which allows for maximizing follow-up rates, as well as the sustainability of the project. The patient cohort covers a wide disease spectrum, in particular early stage CCA, whereas current practice almost always encounters patients at the advanced, incurable stage. All CCA patients in the cohort were histologically confirmed. All major hospitals in the northeast region are actively involved in the program and treatment is based on the routine medical care regime of each hospital. This allows not only sustainability, but also assessment of the effectiveness of the various treatment protocols. The current disease staging practice can then be evaluated and revised.

The sustainability of the project is promising. Intensive efforts by CASCAP to raise the awareness of CCA, after this usually fatal disease had been neglected for more than three decades, have been successful. In December 2014, a year after the initiation of CASCAP, combatting liver fluke infection and cholangiocarcinoma were officially declared to be part of the national health policy. All health care units, the local municipality and community are actively involved in the screening program. Activities under this theme have a high level of support from the National Health Security Office. In addition, CASCAP has established international collaborations with many institutes, including Imperial College in England, the German Cancer Research Foundation, the National University of Singapore, the Royal Veterinary Institute and Karlsruhe Institute of Technology, as well as international funding agencies such as the Wellcome Trust, Center for Global Health and the German Federal Ministry of Education and Research. In an international context, active involvement has begun within the diagnostic and training program. For instance, hospitals and care units in Laos PDR has established an initial collaboration. CASCAP is also embedded in the Cholangiocarcinoma Foundation of Thailand and collaborates with The Alan Morement Memorial Fund (AMMF), the only charity body for CCA in England.

The current weaknesses of the CASCAP include the fact that the awareness of the health risk in participants people and patients is still limited. Based on the perception of the fatal outcome of CCA for late stage patients (“you have to die of something anyway”) is common. In addition, a large proportion of the at risk population are not aware that they are in fact at risk and do not know where they can receive assistance e.g. screening and treatment. A large proportion of the community, the health care providers and doctors do not fully understand that CCA is a curable disease is treated at an early stage.

### Data archival

Data is being collected using a web-based application at www.cascap.in.th. A software package “CASCAP Tools” has been designed for this web application. This is available to health and medical professionals throughout the region. Health care facilities are welcome to freely apply for an account. Health care personnel at the village level enroll at risk populations under their responsible health service area into CASCAP Tools. This allows them to monitor, at any time, whether their participants had undergone U/S monitoring, had been diagnosed, treated, and the clinical outcome until the end of life. This is made possible using the 13-digit citizen identification number with the consent of the individual participants. However, only authorized health personnel can access to the data. General users can access only the summary report without being able to view the individual records. Researchers are welcome to use the individual records that made available under permission of the CASCAP Database Committee. They can request the data by submitting a Data Analysis Plan Proposal at http://www.cascap.in.th/damus/analysis_plan.php.

## Abbreviations

CCA: Cholangiocarcinoma
CASCAP: Cholangiocarcinoma Screening and Care Program
PDF: Periductal fibrosis
CT: Computed Tomography
MRI: Magnetic resonance imaging
AMMF: The Alan Morement Memorial Fund
